# 特发性肺纤维化合并非小细胞肺癌的致病机制及潜在治疗药物研究进展

**DOI:** 10.3779/j.issn.1009-3419.2022.101.45

**Published:** 2022-10-20

**Authors:** 婷 肖, 嘉丽 暴, 湘宁 刘, 慧 黄, 红刚 周

**Affiliations:** 1 300350 天津，南开大学药学院 College of Pharmacy, Nankai University, Tianjin 300350, China; 2 100730 北京，北京协和医院内科 Department of Medicine, Peking Union Medical College Hospital, Chinese Academy of Medical Sciences & Peking Union Medical College, Beijing 100730, China; 3 100730 北京，北京协和医院呼吸与危重症医学科 Department of Respiratory Medicine, Peking Union Medical College Hospital, Chinese Academy of Medical Sciences & Peking Union Medical College, Beijing 100730, China

**Keywords:** 特发性肺纤维化, 肺肿瘤, 致病机制, 治疗药物, Idiopathic pulmonary fibrosis, Lung neoplasms, Pathogenesis, Therapeutic drug

## Abstract

特发性肺纤维化（idiopathic pulmonary fibrosis, IPF）是病因不明的慢性进行性纤维化性间质性肺疾病，IPF也被认为是肺癌的独立风险因素之一，可增加7%-20%的LC发病风险。IPF合并LC，尤其是非小细胞肺癌（non-small cell LC, NSCLC）的发病率逐渐升高，但尚无统一的管理和治疗共识。IPF与NSCLC有着相似的病理特征，均在肺的周围区域出现，在IPF合并NSCLC的患者中，NSCLC往往从IPF的蜂窝区域发展而来，但IPF诱发NSCLC的机制仍不清楚。此外，IPF和NSCLC具有相似的遗传、分子和细胞过程以及常见的信号转导通路，靶向IPF和NSCLC共同的信号通路将成为IPF合并NSCLC的潜在治疗药物。本文就针对共同参与IPF和NSCLC的主要分子机制以及靶向这些信号通路的在研药物的研究进展进行综述。

## Abstract (en)

Idiopathic pulmonary fibrosis (IPF) is a chronic progressive fibrous interstitial lung disease of unknown etiology. IPF is also considered to be among the independent risk factors for lung cancer, increasing the risk of lung cancer by 7% and 20%. The incidence of IPF complicated with lung cancer, especially non-small cell lung cancer (NSCLC), is increasing gradually, but there is no consensus on unified management and treatment. IPF and NSCLC have similar pathological features. Both appear in the surrounding area of the lung. In pathients with IPF complicated with NSCLC, NSCLC often develops from the honeycomb region of IPF, but the mechanism of NSCLC induced by IPF remains unclear. In addition, IPF and NSCLC have similar genetic, molecular and cellular processes and common signal transduction pathways. The universal signal pathways targeting IPF and NSCLC will become potential therapeutic drugs for IPF complicated with NSCLC. This article examines the main molecular mechanisms involved in IPF and NSCLC and the research progress of drugs under development targeting these signal pathways.

特发性肺纤维化（idiopathic pulmonary fibrosis, IPF）是一种病因不明的罕见慢性进行性纤维化间质性肺炎，其生存率甚至低于某些癌症，自诊断时起中位生存期仅有2年-4年。尽管IPF的病因不明，但我们已经知道吸烟或二手烟、年龄大、职业暴露相关的环境因素被认为是增加IPF患者发病和进展的可能性风险因素。IPF的发生和进展会导致肺结构和生物学特性的巨大改变，使肺泡气体交换受损，肺功能下降，最终导致呼吸衰竭。目前仅有两种上市的抗肺纤维化药物—吡非尼酮和尼达尼布，它们一定程度上能够延缓IPF患者的疾病进展并延长生存期，但无法逆转肺纤维化。肺癌是全球发病率和死亡率最高的癌症类型，约85%的肺癌为非小细胞肺癌（non-small cell lung cancer, NSCLC）。早在1965年就有人提出IPF和肺癌之间存在一定的联系。近些年，人们发现吸烟史、高龄、男性等可能是IPF患者发生肺癌，主要是NSCLC的重要危险因素，此外，IPF本身也被认为是一种诱导肺癌发生的独立危险因素，被视为癌前病变。IPF人群中肺癌的发生率也逐渐升高，据报道，IPF合并肺癌的发生率为2.8%-48%，IPF患病3年后的累积肺癌发病率超过80%。肺癌通常发生于IPF患者的肺外周区域，与蜂窝状相关病变部位相重合，从蜂窝区域或在蜂窝和非纤维化区域之间的边界发展。最近在IPF合并肺癌方面的研究集中在识别IPF和肺癌之间的共同分子途径及寻找有效的治疗手段，以便更好地管理和治疗这两种疾病的患者。本文主要通过分析两种疾病共同的分子靶点和分子机制，总结几个主要信号通路在IPF和NSCLC中的作用机制，归纳靶向这些信号通路的在研药物，以了解这些可能有助于IPF合并NSCLC患者治疗的潜在方法。

## 1 IPF和NSCLC的共同致病机制分析

IPF和NSCLC之间存在很多相同或相似的分子机制或疾病特点，如肺纤维化中存在肌成纤维细胞的活化和增殖，LC中癌症相关成纤维细胞的激活和增殖也能促进LC的进展，IPF和NSCLC疾病进展中都存在一些生长因子、细胞因子和细胞外基质蛋白的改变等。为了进一步明确IPF和NSCLC之间可能存在共同的分子靶点和机制，我们利用GeneCards数据库检索了IPF和NSCLC的分子靶点，IPF相关分子靶点3,339个，NSCLC相关分子靶点4,117个，对两种疾病的靶点进行维恩分析后有1,584个共同的分子靶点。随后我们对这些共同的分子靶点进行了KEGG富集分析，这些分子靶点主要参与了PI3K/AKT/mTOR信号通路、细胞因子及JAK/STAT信号通路、凝血级联和钙离子信号通路、炎症免疫相关信号通路以及癌症、感染相关通路，还涉及细胞的凋亡、衰老、自噬、黏附、细胞周期、耐药等细胞生物学功能。本文主要针对几个常见信号通路在IPF和NSCLC中的作用机制及针对IPF-NSCLC的潜在治疗药物进行总结和探讨。

## 2 IPF-NSCLC中的关键信号通路及潜在治疗药物

### 2.1 PI3K-AKT-mTOR信号通路

#### 2.1.1 PI3K-AKT-mTOR信号通路在IPF中的功能

PI3K-AKT-mTOR信号通路参与调节细胞生长、运动、增殖、代谢和存活等多种生物学功能，是细胞的核心信号通路之一^[1]^。PI3K家族可分为Ⅰ、Ⅱ、Ⅲ三类，Ⅰ类PI3K包含PI3Kα、PI3Kβ、PI3Kδ和PI3Kγ四种亚型^[2]^，其中PI3Kα和PI3Kγ通常在人肺成纤维细胞中表达且在肺部疾病中上调或突变^[3]^，PI3Kγ通常在IPF患者的肺成纤维细胞中过度表达^[4]^。AKT可被上游的PI3K蛋白激活发生磷酸化。丝氨酸/苏氨酸蛋白激酶AKT也包含三个成员，分别是AKT1、AKT2和AKT3。AKT1和AKT2亚型主要参与调控肺纤维化。AKT1在肺纤维化中可通过调节线粒体来增强肺泡巨噬细胞的凋亡抗性，而AKT2的缺失可阻碍博来霉素诱导的小鼠肺纤维化和炎症的发生^[5]^。PI3K-AKT-mTOR信号通路参与肺纤维化发生发展的多个阶段。在肺泡上皮细胞损伤后，PDGFR的活化可激活PI3K/AKT进而调控肺上皮细胞的凋亡和衰老以及上皮细胞向间充质转化（epithelial to mesenchymal transition, EMT）过程。在肺成纤维细胞中PI3K/AKT可以被Wnt和转化生长因子-β（transforming growth factor-β, TGF-β）通路共激活并调控下游mTOR、缺氧诱导因子-1α（hypoxia-inducible factor-1α, HIF-1α）以及FOX家族的表达或活化进而上调肺纤维化中α-SMA的表达和肌成纤维细胞的活化促进肺纤维化的发生和进展^[6]^。由于PI3K-AKT-mTOR信号通路在调控肺纤维化进展中发挥着重要作用，因此，靶向PI3K-AKT-mTOR信号通路将成为治疗IPF的药物开发的新策略。

#### 2.1.2 PI3K-AKT-mTOR信号通路在NSCLC中的作用

自PI3K在肿瘤中的功能被发现以来，PI3K信号通路的激活已被证实是调控癌症发展的核心。在NSCLC中，PI3K-AKT-mTOR通路也在调控肿瘤的发生和进展中发挥重要作用^[7]^。在NSCLC患者中常见PIK3CA的体细胞突变和异常扩增。在一项针对1,144例NSCLC患者的二代测序研究^[8]^中发现，3.7%的患者存在*PIK3CA*的突变，其中肺鳞癌患者占8.9%，肺腺癌患者有2.9%。日本研究者^[9]^对86种NSCLC细胞系和356个NSCLC患者的肿瘤组织进行了*PIK3CA*突变和扩增的检测，细胞系中*PIK3CA*突变和扩增的比例占12.8%，而肿瘤组织中的突变和扩增比例占19.1%，这项研究中同样发现肺鳞状细胞癌（33.1%）的*PIK3CA*的扩增比例高于肺腺癌（6.2%）。*PIK3CA*的突变或扩增能够增强AKT的活性。在大多数的NSCLC患者中存在AKT的活性增强，在一项研究^[10]^中研究者通过免疫组化的方法检测了NSCLC肿瘤组织中AKT的表达，结果显示，51%的肿瘤组织中存在AKT的高表达。AKT活性的增加还与AKT通路的激活和mTOR的上调显著相关。作为PI3K-AKT的下游通路，在NSCLC肿瘤中mTOR的磷酸化水平也显著升高，约有90%的肺腺癌患者、60%的大细胞癌患者和40%的鳞状细胞癌患者mTOR的磷酸化水平升高^[11]^。此外，还有研究^[12]^表明，PI3K/AKT/mTOR信号通路的激活与表皮生长因子受体（epidermal growth factor receptor, *EGFR*）的突变相关，表明PI3K/AKT通路的激活可能会导致NSCLC患者对EGFR抑制剂产生获得性耐药。因此，对于NSCLC的治疗来说，PI3K-AKT-mTOR信号通路也是一个有潜力的治疗靶点。

**图 1:**
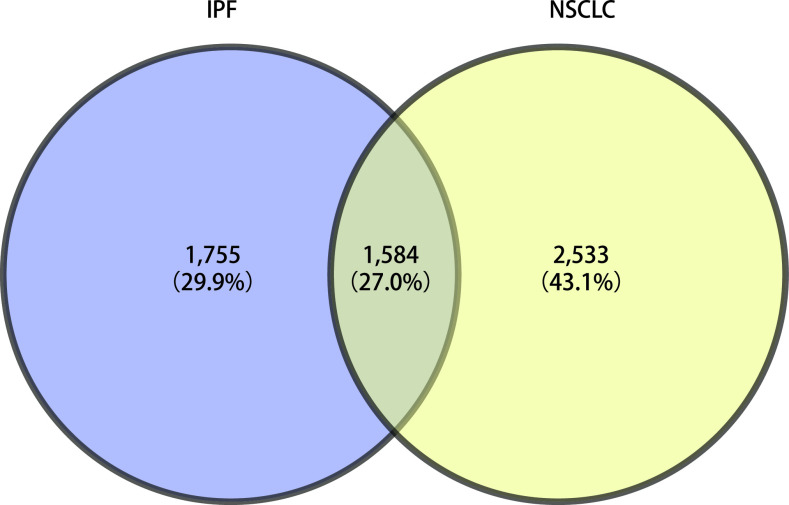
IPF和NSCLC分子靶点的维恩分析 Venn analysis of IPF and NSCLC molecular targets. IPF: idiopathic pulmonary fibrosis; NSCLC: non-small cell lung cancer.

#### 2.1.3 靶向PI3K-AKT-mTOR信号通路的潜在IPF-NSCLC治疗药物

目前已有许多PI3K、AKT和mTOR的特异性抑制剂正在开发中，并且有一些抑制剂已经分别在NSCLC和IPF的治疗上进入临床试验阶段，大多数的PI3K-AKT-mTOR通路抑制剂仅在NSCLC或IPF一种疾病中开展了临床前或临床试验阶段的研究，但仍有少数药物在NSCLC和IPF的治疗中均有报道。PX-886是一种泛PI3K抑制剂，在一项PX-886联合多西他赛的Ⅰ期临床试验^[13]^中，1例*PIK3CA*突变的NSCLC患者在停用多西他赛后对继续使用PX-866的反应延长。一项临床前的研究^[14]^报道了PX-866可以在体内抑制转化生长因子-α（transforming growth factor-α, TGF-α）诱导的肺纤维化的进展。雷帕霉素，也称为西罗莫司，是一种口服的mTOR抑制剂，在与阿法替尼联用的临床试验中显示出西罗莫司显著的毒性，在与化疗药物培美曲塞的联合用药的临床试验中西罗莫司则显示出了潜在的抗肿瘤活性。随着研究的深入，人们发现mTOR靶点在肺纤维化的治疗中也尤为重要。因此，mTOR抑制剂也被用于肺纤维化的临床前研究。已报道，mTOR抑制剂雷帕霉素可改善博来霉素诱导的小鼠肺纤维化，并可以预防和抑制由TGF-α的表达和增强EGFR信号转导引起的进行性肺纤维化的进展^[15]^。一项雷帕霉素治疗肺纤维化的临床试验研究^[1]^正在进行中，研究结果尚未公开。但在另一项肺癌治疗的临床研究^[16]^中观察到与雷帕霉素相关的3级或4级不良反应。PX-866和雷帕霉素将来可能成为IPF合并NSCLC的潜在治疗药物，其有效性还需更多的临床数据的支持，且他们可能造成的不良反应和危险因素不容忽视。

### 2.2 JAK-STAT信号通路

JAK-STAT信号通路主要包含胞外配体、配体的结合受体、JAK激酶以及STAT家族蛋白四个部分，胞外配体通常是细胞因子、生长因子或干扰素，JAK激酶家族由JAK1、JAK2、JAK3和TYK2组成，STAT家族则包含STAT1、STAT2、STAT3、STAT4、STAT5a、STAT5b和STAT6共7个成员。JAK-STAT通路的激活起始于胞外配体与受体的结合，诱导受体二聚化，受体的活化可使JAK磷酸化，磷酸化的JAK进一步磷酸化STAT蛋白，磷酸化的STAT蛋白可形成同源二聚或异源二聚体并转位至细胞核内发挥功能。JAK-STAT信号通路的激活可调控对细胞稳态至关重要的多个细胞过程，进而参与癌症、炎症、自身免疫性疾病和肺纤维化等疾病的进展。

#### 2.2.1 JAK-STAT信号通路与肺纤维化

JAK-STAT信号通路异常激活以及关键蛋白的过表达能够促进肺纤维化的疾病进程。已有报道证明，在博来霉素诱导的肺纤维化模型的肺组织中存在JAK1、JAK2、STAT1和STAT3等蛋白的过表达或磷酸化水平升高。JAK1和STAT1主要定位于博来霉素诱导的肺纤维化小鼠的炎症细胞和肺上皮细胞中，在肺纤维化小鼠的肺组织中可检测到JAK1和STAT1蛋白及其活性形式的高表达^[17]^。在IPF患者中，JAK2主要分布于增生的Ⅱ型肺泡上皮细胞、成纤维细胞、内膜和小肺动脉中膜，且JAK2及其磷酸化形式在IPF患者的肺组织中的表达均明显增加^[18]^。STAT3主要在博来霉素诱导的肺纤维化小鼠的肺泡巨噬细胞、内皮细胞和中性粒细胞中分布较高，其磷酸化形式也存在于肌成纤维细胞和肺泡巨噬细胞中^[19]^。在IPF患者中，STAT3存在于小肺动脉、增生的肺泡细胞和成纤维细胞中，在IPF患者的致密纤维化区域的肺动脉细胞、肌成纤维细胞和肺泡上皮细胞的细胞核中可检测到高水平的p-STAT3^[20]^。肺纤维化中JAK-STAT信号通路的异常激活参与调控肺纤维化进展的多个生物学过程，包括促进成纤维细胞向间充质转化（fibroblast to mesenchymal transition, FMT），促进EMT，促进肺泡上皮细胞的衰老和凋亡及抑制肌成纤维细胞的凋亡和自噬等。JAK-STAT通路在肺纤维化中的重要性使其成为肺纤维化治疗上一个有吸引力的目标，靶向JAK-STAT信号通路治疗肺纤维化将在未来的临床研究中得到证明。

#### 2.2.2 JAK-STAT信号通路与NSCLC

JAK-STAT通路的异常与包括LC在内的多种肿瘤的发生密切相关，参与肿瘤的增殖分化、细胞凋亡、免疫逃逸、血管新生、癌细胞干性、耐药性和侵袭转移等多个过程。STAT3的表达与NSCLC的组织分化程度和淋巴结转移有关，已有研究证实在NSCLC的肿瘤组织中STAT3的表达相较于癌旁组织明显升高，低分化及有淋巴结转移的肿瘤组织中STAT3的表达也高于高分化和无淋巴结转移组。还有研究^[21]^发现，STAT3的磷酸化与EGFR的异常活化和表达呈正相关，这也与肿瘤的生长和侵袭能力具有一定的相关性。STAT3的磷酸化与肿瘤细胞的凋亡呈负相关，JAK-STAT通路的激活可上调下游抗凋亡蛋白和增殖相关蛋白的过度表达，从而增强肺癌细胞的抗凋亡能力和增殖能力。近年来，JAK-STAT3通路已经成为肺癌靶向治疗的新靶点，为LC的治疗提供了新的方向。

#### 2.2.3 靶向JAK-STAT信号通路的IPF-NSCLC潜在治疗药物

靶向JAK-STAT信号通路的抑制剂主要包括激活配体的抑制剂、配体相关受体的抑制剂、JAK抑制剂以及STAT抑制剂等。目前也不乏有靶向该通路的药物已经上市，如IL-6单克隆抗体司妥昔单抗和托珠单抗以及JAK抑制剂托法替尼、巴瑞替尼和鲁索替尼等。然而，目前报道的同时对肺纤维化和LC有治疗效果的抑制剂或药物仍然非常有限。鲁索替尼是一种已上市的JAK1/2选择性抑制剂。在NSCLC的临床前研究中已经证明鲁索替尼能够通过抑制STAT3的活化，恢复肺癌细胞对顺铂化疗的敏感性，增强细胞的凋亡并抑制肿瘤生长。鲁索替尼治疗NSCLC患者的临床结果喜忧参半。在鲁索替尼联合培美曲塞与顺铂以及鲁索替尼联合厄洛替尼的临床研究中均未展现出临床益处或未达到疗效终点^[22]^。但在一项鲁索替尼联合阿法替尼的IB期临床研究^[23]^中，有23%的患者实现了部分缓解，疾病控制率达到93%，且耐受性良好。尽管还没有鲁索替尼在肺纤维化方面的临床研究，但在博来霉素诱导的小鼠肺纤维化模型中使用鲁索替尼可显著改善小鼠的肺纤维化病变并降低纤维化分子标志物的水平^[24]^。一种靶向STAT3的SH2结构域的小分子抑制剂C188-9已被报道可通过靶向抑制STAT3的磷酸化下调下游抗凋亡蛋白Bcl-2、Bcl-xL和增殖相关蛋白Survivin和Cyclin的表达，抑制肺癌细胞A549的增殖和诱导细胞凋亡，进而抑制小鼠肿瘤的生长^[25]^。在肺纤维化中也有报道称C188-9能够抑制STAT3并促进BLM诱导的纤维化小鼠模型中纤维化标志物的减少，并可减弱TGF-β诱导的肺纤维化小鼠的原代肺成纤维细胞的FMT^[26]^。JSI-124也称葫芦素Ⅰ，是从葫芦科植物中分离出的一种三萜类分子，已有研究^[27]^表明其可通过抑制EKR激活及mTOR和STAT3的磷酸化诱导肺癌A549细胞的凋亡和自噬。在肺纤维化的研究中，JSI-124可对p-JAK2和p-STAT3产生双重抑制从而降低肺成纤维细胞的FMT和肺泡上皮细胞的EMT，并且能够在BLM诱导的大鼠纤维化中抑制纤维化标志物的表达^[28]^。尽管上述抑制剂分别在LC和肺纤维化中显示出了治疗作用，但其在肺纤维化合并LC患者中的治疗作用尚需更多的临床评估来证实。

### 2.3 凝血级联与蛋白酶激活受体

#### 2.3.1 凝血级联与蛋白酶激活受体在肺纤维化中的作用

在肺纤维化疾病发生发展的早期阶段，肺组织损伤后会激活凝血级联反应，促发血小板的活化，随后局部分泌可溶性介质，导致血管通透性增加。血液中的促凝血因子（如TF、FVII、FXa和凝血酶等）可激活肺成纤维细胞或上皮细胞中的PARs受体促进肺成纤维细胞向肌成纤维细胞转变，细胞外基质的积累以及肺上皮细胞的EMT。已有报道，FXa以PAR1依赖的方式诱导肌成纤维细胞分化和TGF-β活化，还可通过PAR2诱导成纤维细胞的促纤维化作用。FVIIa则可通过PAR2刺激人肺成纤维细胞的增殖和ECM产生。凝血酶等促凝血因子还可以诱导几种促纤维化因子的分泌，包括TGF-β、结缔组织生长因子（connective tissue growth factor, CTGF）、血小板衍生生长因子（platelet-derived growth factor, PDGF）、单核细胞趋化蛋白-1和白细胞介素-6（interleukin-6, IL-6）等。因此，上皮损伤引起的凝血激活已经逐渐被认为是纤维化肺疾病发病机制的关键因素。此外，与普通人相比，IPF患者更容易出现高凝状态，具有这种血栓前状态的患者疾病的严重程度更高，死亡风险增加^[29]^。凝血因子及其受体在IPF中的潜在重要性进一步强调了靶向该机制对于开发治疗肺纤维化药物的潜力。

#### 2.3.2 凝血级联与蛋白酶激活受体在NSCLC中的作用

早在一个多世纪以前人们就发现凝血级联引起的静脉血栓形成与癌症的进展密切相关，血栓形成与癌症的相关患病率为10%-20%。凝血酶通过激活血小板还可促进肿瘤聚集、黏附、生长、转移和血管生成，从而导致肿瘤的恶性程度增加^[30]^。凝血酶通过激活PAR1受体在肿瘤的发生、炎症和转移扩散中发挥重要作用。Zhao等^[31]^的研究发现，凝血酶通过PAR1介导的核因子κB（nuclear factor kappa-B, NF-κB）信号级联反应诱导NSCLC细胞VM的形成，而这一作用在PAR1敲除的细胞中被消除。且凝血酶、PAR1的表达水平与NSCLC患者的预后密切相关。另有研究^[32]^报道，依诺肝素可通过靶向抑制PAR1干扰其下游的两条主信号通路MAPK/ERK和PI3K/AKT的激活抑制NSCLC的增殖和迁移，因此依诺肝素也可能有望作为传统LC化疗的辅助手段。PAR2也是PARs家族的主要成员之一，其激活配体为胰蛋白酶等，已有研究^[33]^表明，抑制PAR2的活化可减弱ERK介导的NSCLC EMT和PD-L1的表达从而逆转奥希替尼耐药。PAR2在肺腺癌中还可促进Slug介导的EMT而诱导肺腺癌细胞的迁移^[34]^。Wu等^[35]^的研究还证明PAR2启动子的低甲基化可上调*PAR2*基因的表达进而促进肺腺癌的进展。因此，靶向凝血酶及PARs也是LC治疗的有潜力的靶点。值得注意的是，不少研究还发现TGF-β/ΑLΚ5和PAR1/PAR2之间存在信号串扰，既可调控纤维化的进展也能够控制癌症的恶性演进。

#### 2.3.3 靶向蛋白酶激活受体的IPF-NSCLC潜在治疗药物

目前针对凝血级联及PARs家族的药物并不是很多。靶向凝血酶的已上市药物有华法林和达比加群等，尽管有研究表明达比加群可以抑制肺成纤维细胞的增殖、胶原沉积和ECM形成^[36]^，但尚未有达比加群治疗LC方面的研究。已上市的PAR1抑制剂仅有Vorapaxar一种药物，此外还有一些临床前研究阶段的PAR1抑制剂，仅P1pal-12S报道了其在博来霉素诱导的小鼠肺纤维化中的作用^[37]^。PAR2抑制剂尚无已上市药物，但Lin等^[38]^的研究报道了一种PAR2抑制剂P2pal-18S在博来霉素诱导的小鼠肺纤维化中的作用。PAR2可以通过反式激活EGFR诱导EGFR对吉非替尼等靶向药物的耐药，而PAR2抑制剂P2pal-18S则通过抑制PAR2的激活增加肺癌细胞对吉非替尼的敏感性，逆转肺癌细胞耐药^[39]^。由此可见，PAR2抑制剂未来可能是IPF-LC治疗的潜在药物。

### 2.4 免疫检查点[程序性死亡分子-1（programmed cell death-1, PD-1）及其主要配体（programmed cell death ligand 1, PD-L1）]

#### 2.4.1 IPF中的免疫检查点

免疫检查点在肿瘤细胞的免疫逃逸中至关重要，IPF与癌症在致病机制上有一定的重叠，免疫检查点在IPF中的潜在作用也逐渐被发现。在一项比较IPF患者与健康对照者的可溶性PD-L1血清水平的初步研究^[40]^中，在IPF患者中观察到PD-L1的表达增加了3倍。而对IPF肺组织换件的免疫组化分析中也发现，12例患者中的9例患者肺组织中可检测到PD-L1的表达。也有研究发现，与健康对照组相比，IPF患者外周血中PD-L1的表达未增加，但外周血和肺组织中T淋巴细胞的PD-1表达显著增加。CD4^+^ T细胞上的PD-1表达导致STAT3上调和随后的IL-17A和TGF-β的表达，而阻断PD-1/PD-L1轴可下调CD4^+^ STAT3介导的IL-17A和TGF-β的产生。另外，一项研究^[41]^还发现，PD-L1在侵袭性成纤维细胞上的表达也可促进肺纤维化。对成纤维细胞基因敲除PD-L1或用PD-L1抗体处理可显著抑制成纤维细胞的侵袭、迁移和体内胶原蛋白的产生。这些研究都证明了IPF中PD-1和PD-L1的异常表达和免疫调控对IPF疾病进展的影响。

#### 2.4.2 NSCLC中的免疫检查点

在肿瘤免疫微环境中，T细胞需要启动活化并识别肿瘤细胞，T细胞的活化需要肿瘤细胞和抗原呈递细胞（antigen presenting cell, APC）表面的主要组织相容性复合体（major histocompatibility complex, MHC）受体与T细胞表面的T细胞受体（T cell receptor, TCR）结合以及APC表面的CD28与T细胞表面的CD28结合。PD-1是T细胞增殖和功能的抑制剂，在免疫耐受的生理维持以及肿瘤检测中起着至关重要的作用。而在肿瘤细胞中存在PD-L1蛋白的表达上调，肿瘤细胞表面的PD-L1可与T细胞表面的PD-1蛋白结合后抑制T细胞TCR的下游信号传导，干扰了MHC呈递抗原提供的刺激信号，从而抑制T细胞的活化、增殖和细胞因子的产生，最终导致细胞凋亡，使T细胞无法发动有效的免疫反应而衰竭。因此，抑制PD-1/PD-L1相互作用可以有效地挽救“耗尽”的T细胞的活性，促进抗原表达细胞对它们的激活^[42]^。

#### 2.4.3 免疫检查点抑制剂在IPF-NSCLC治疗中的应用

目前免疫疗法已经成为LC治疗的一种新的治疗选择，免疫检查点抑制剂也成为最常用的免疫疗法药物，PD-1/PD-L1免疫检查点抑制剂，如PD-1单克隆抗体纳武利尤单抗（Nivolumab）、帕博利珠单抗（Pembrolizumab）和PD-L1单克隆抗体阿替利珠单抗（Atezolizumab）等已广泛应用于晚期/转移性和局部晚期NSCLC患者，临床数据也显示出它们对生存和预后的显著改善^[43]^。目前虽尚未有免疫检查点抑制剂直接应用于临床IPF患者的治疗，但临床前的研究表明，PD-1/PD-L1抑制剂可显著减轻二氧化硅诱导的小鼠肺纤维化。值得注意的是，在一项病例回顾性研究^[44]^中报道了利用纳武单抗治疗IPF合并肺腺癌患者的案例，该患者在初始化疗失败后，接受了纳武利尤单抗治疗，并获得了完全的缓解且没有任何IPF恶化的迹象，对纳武利尤单抗的持续反应时间超过1年。在一项纳武利尤单抗治疗肺鳞癌合并IPF的病例报告^[45]^中，患者接受纳武利尤单抗治疗8个月未显示出明显的不良反应，且疾病得到稳定控制。这些成功的案例提示了免疫检查点抑制剂将是IPF-LC的一种有希望的治疗选择。但是，在LC的治疗中免疫检查点抑制剂的应用一定程度上提高了患者免疫检查点抑制剂相关肺炎的发生率，因此其对IPF急性加重风险的增加仍不容忽视。

## 3 展望

流行病学数据显示，吸烟、男性、高龄等是IPF和LC共同的致病因素，而IPF现也被认为是诱发LC的独立危险因素，通常在IPF患者紧邻肺纤维化的区域外周发展为肺肿瘤，IPF合并LC尤其是NSCLC的发病率也越来越高。然而，目前对于IPF合并LC的诊断、管理和治疗尚无统一的共识，临床中，常根据患者情况采取不同的治疗方法，如手术、化疗、靶向治疗及抗纤维化治疗等，但目前的LC治疗手段可能会增加IPF的急性加重的风险，尚无有效、公认的治疗手段，IPF合并LC患者的治疗是目前临床面临的主要困境之一。研究表明IPF和LC的致病因素和机制存在许多相似性，包括相似的遗传改变和生物标志物以及共同的常见信号转导通路的异常激活。基于IPF和LC之间共同的分子机制，靶向这些共同的信号通路药物将成为抗癌和抗肺纤维化的多效药物，可能对IPF合并LC患者具有潜在的治疗作用。然而，目前还缺乏合理的临床前IPF-NSCLC的药效评价方法来评价这些靶向共同信号通路的潜在多效药物对IPF-NSCLC的效果。因此，将来亟需开发合适的IPF-NSCLC动物模型以及开展更多的临床研究来发现IPF-NSCLC的治疗新策略。
